# *Sous-Vide* as a Technique for Preparing Healthy and High-Quality Vegetable and Seafood Products

**DOI:** 10.3390/foods9111537

**Published:** 2020-10-25

**Authors:** Sandra Zavadlav, Marijana Blažić, Franco Van de Velde, Charito Vignatti, Cecilia Fenoglio, Andrea M. Piagentini, María Elida Pirovani, Cristina M. Perotti, Danijela Bursać Kovačević, Predrag Putnik

**Affiliations:** 1 Department of Food Technology, Karlovac University of Applied Sciences, Trg J. J. Strossmayera 9, 47 000 Karlovac, Croatia; marijana.blazic@vuka.hr; 2 Facultad de Ingeniería Química, Instituto de Tecnología de Alimentos, Universidad Nacional del Litoral (FIQ, UNL), 1º de Mayo 3250, Santa Fe 3000, Argentina; francovandevelde@hotmail.com (F.V.d.V.); cvignatti@fiq.unl.edu.ar (C.V.); cfenoglio@gmail.com (C.F.); ampiagen@fiq.unl.edu.ar (A.M.P.); mpirovan@fiq.unl.edu.ar (M.E.P.); 3 Consejo Nacional de Investigaciones Científicas y Técnicas (CONICET), Santiago del Estero 2829, Santa Fe 3000, Argentina; cperotti@fiq.unl.edu.ar; 4 Facultad de Ingeniería Química, Instituto de Lactología Industrial (INLAIN), Universidad Nacional del Litoral (FIQ, UNL/CONICET), Santiago del Estero 2829, Santa Fe 3000, Argentina; 5 Faculty of Food Technology and Biotechnology, University of Zagreb, Pierottijeva 6, 10000 Zagreb, Croatia; dbursac@pbf.hr; 6 Greenbird Medical Inc., Trg dr. Žarka Dolinara 18, 48 000 Koprivnica, Croatia; 7 Department of Food Technology, University North, Trg dr. Žarka Dolinara 1, 48000 Koprivnica, Croatia

**Keywords:** *sous-vide* cooking, vegetables, seafood, cephalopods, safety, nutritive quality

## Abstract

*Sous-vide* is a technique of cooking foods in vacuum bags under strictly controlled temperature, offering improved taste, texture and nutritional values along with extended shelf life as compared to the traditional cooking methods. In addition to other constituents, vegetables and seafood represent important sources of phytochemicals. Thus, by applying *sous-vide* technology, preservation of such foods can be prolonged with almost full retention of native quality. In this way, *sous-vide* processing meets customers’ growing demand for the production of safer and healthier foods. Considering the industrial points of view, *sous-vide* technology has proven to be an adequate substitute for traditional cooking methods. Therefore, its application in various aspects of food production has been increasingly researched. Although *sous-vide* cooking of meats and vegetables is well explored, the challenges remain with seafoods due to the large differences in structure and quality of marine organisms. Cephalopods (e.g., squid, octopus, etc.) are of particular interest, as the changes of their muscular physical structure during processing have to be carefully considered. Based on all the above, this study summarizes the literature review on the recent *sous-vide* application on vegetable and seafood products in view of production of high-quality and safe foodstuffs.

## 1. The Perspective of the *Sous-Vide* Technique

*Sous-vide* is a professional cooking technology that finds its application in home, catering, molecular gastronomy and food industry, also known as lapping, vacuum cooking, vacuum-packed cooking or baking-cooling with vacuum packages [1,2,3]. On the one side, *sous-vide* has been demonstrated as the secret of great chefs worldwide for decades and, on the other side, this technique was the result of increased customer demand for “fresh-like” and good-quality processed foods [4,5].

*Sous-vide* means cooking under vacuum and includes a process where raw foods or half-cooked foods are placed in a plastic pouch or bag, hermetically sealed, and cooked slowly in a water bath at 65–95 °C over prolonged time (usually from 1 to 7 h). However, this can take up to 48 h or even more for some foods. With this method, native food juiciness can be retained while avoiding overcooking [6]. Among many advantages, it is important to point out that *sous-vide* cooking requires low-cost operations and equipment to provide consumers with high-quality ready-to-eat food products [7].

*Sous*-*vide* foods can be classified into groups based on the magnitude of applied heat treatment [2,8]. For instance, low temperature and longtime cooking method (LTLT) results in foods with favorable characteristics such as enhanced flavor and aroma, increased tenderness and desirable texture, reduced lipid oxidation which leads to extended shelf life, reduced losses of (volatile) flavor (due to vacuum packaging) and improved color and visual appeal [9,10,11,12]. Although the nutritional content in *sous-vide* cooked food is maintained and no additives or preservatives are required, this cooking technique modifies nutrients (e.g., proteins) in order to improve functionality [13].

At the point of reaching the optimal internal temperature and the desired textural properties, the food is quickly cooled and then stored refrigerated or frozen until the time of serving, regardless of whether it refers to restaurants or domestic use [6,14,15]. 

The assessment of the microbial safety is of great importance for this culinary method; therefore, it is essential to know the effects of such treatments on the microorganisms for evaluating products’ safety [14]. Several studies have shown that the presence of pathogens in *sous-vide* foods at the time of consumption originates from the raw materials as they survive cooking [16]. The optimal temperature for the growth of the most pathogenic bacteria is between 30 and 50 °C, where inhibition of bacterial reproduction and growth starts. Thus, the core temperature of food during processing should not fall below 54.4 °C and cooking must be held for up to 6 h to ensure inactivation of the food pathogens such as *Salmonella* species, *Listeria monocytogenes*, and the pathogenic strains of *Escherichia coli*. This is a critical point only in case the food is not previously pasteurized.

For the case when the *sous-vide* product needs to be frozen for further use, freezing must be conducted rapidly and immediately after the treatment to prevent or limit possible health hazards. Besides, storage of *sous-vide* food in vacuum pouches is appropriate for preventing recontamination [6,8]. Considering safety, special attention is given to the toxicity derived from the spore-forming pathogenic bacteria that are capable to withstand the mild heat treatments, and hence able to germinate during refrigeration.

For foods with a shelf life of less than 10 days, it is recommended to cook at 70 °C for 2 min or equivalent to reach a 6-log reduction of the most heat-resistant vegetative pathogen (*Listeria monocytogenes*). In case of foods with a shelf life of more than 10 days, cooking at 90 °C for 10 min is recommended (or equivalent) to reach a 6-log reduction of spores *Clostridium botulinum*. However, nowadays, there is a trend of applying low temperatures in food processing (e.g., from 40 to 70 °C), which are considered as a “danger zone” for microbial growth, thus being difficult to assure product safety [14,15]. 

Stringer et al. [5] thoroughly reported available information about bacterial contamination in the *sous-vide* procedure and compared it to the traditional cooking methods. Heat resistance of pathogens has been investigated extensively at traditional cooking temperatures and powerful mathematical models were used to predict the growth of microorganisms, without the need to test every product and sets of parameters. Nevertheless, there is a lack of data for thermal inactivation of pathogens in low-temperature conditions under vacuum, similar to those applied in *sous-vide* [8,17]. Accordingly, the aim of the current study was to review modern and useful *sous-vide* techniques for preparing healthy and high-quality vegetables and seafood with respect to nutrition and safety. 

## 2. *Sous-Vide* Application for Vegetable Processing

*Sous-vide* is different from minimally processed produce, as according to the International Fresh-cut Produce Association (IFPA), minimally processed vegetables are defined as "any fresh fruit or vegetable or any combination thereof that has been physically altered from its original form, but remains in a fresh state” [18]. Minimally processed vegetables are consumed raw and their processing involves several preparation steps that do not include cooking, nor do these types of products need additional preparations prior to consumption. 

Oppositely, vegetables that have been processed with *sous-vide*, even if they have been treated with a low temperature, are not “fresh” anymore and therefore not minimally processed. The raw vegetables are processed with various operations, such as sorting, peeling, washing, chopping, cutting, blanching or pre-cooking. Then, raw or pre-cooked vegetables are packaged in plastic pouches or bags. Immediately before sealing under vacuum, air is mechanically removed from the package and then thermally processed. Sometimes *sous-vide* is a pasteurization step that reduces the microbial load, nonetheless this is not sufficient to prevent spoilage throughout the shelf life if stored under ambient conditions. When heating temperature is chosen for convectively heated foods, usually at the set temperature, the focus is only given to a length of holding. However, equally important is the time needed for both, to heat and to cool foods at desired temperatures [19]. The challenge is to define cooking conditions suitable to preserve the high quality of vegetables. After heating, vegetables are rapidly cooled down (within 2 h or less) below 3.3 °C, at this point, product temperature should be kept constant during entire storage and distribution. 

The application of *sous-vide* technology has been studied for various vegetables [20,21,22,23,24], but not for fruits. Fruits are generally eaten raw, however chefs sometimes cook apples and pears until they are tender [6]. In the case of vegetables, the low amount of oxygen inside the pack will tend to preserve nutritional value and sensory quality, as compared to the other conventional methods such as boiling, steaming or microwaving. Vegetables that are treated by these methods will tend to lose their nutrients, as cellular walls are damaged by heat, which allows water and nutrients to leak out. The *sous-vide* procedure leaves vegetable cell walls mostly intact and makes food tender by dissolving the cementing material (pectin) that holds the cells together [25]. As pectin begins to dissolve between 82 and 85 °C, its depolymerization is bound to lead to texture degradation that might not be a desired consequence of *sous-vide* treatment [26]. These values, therefore, constitute the lowest viable temperature for the *sous-vide* cooked vegetables [6]. Thus, unlike other products, such as meat, for which the adequate temperature is 65–70 °C, *sous-vide* cooking of vegetables, due to various thermal diffusivity, must be performed at temperatures that are closer to 100 °C in order to inactivate two major foodborne pathogens, *E. coli* and *Salmonella* [21,25].

### 2.1. Changes in Physicochemical Properties and Sensory Quality of Sous-Vide Vegetable Products

Interestingly enough, *sous-vide,* in some cases with nutrient retention, even intensifies characteristic flavors, as it happens with rutabaga and turnip [27]. Additionally, other physical properties of vegetables, mainly texture and color, are greatly influenced by these treatments [28]. Regarding physicochemical properties, after the processing, pH and water activity remain almost the same as in raw vegetables, probably because they are prepared without additives. Even salt addition, as a taste enhancer, does not significantly modify water activity of the product [17]. However, dos Reis et al. [29] reported that pH of broccoli and cauliflower inflorescences diminished half a point after *sous-vide* processing (90 °C, 20 min) when compared to the fresh samples. This is probably due to some cellular ruptures in the walls that released inner acids.

*Sous-vide* treatment may reduce the weight loss of cooked vegetable products. Gonnella et al. [23] reported that asparagus spears’ weight loss was 2.1% after *sous-vide* processing (80 °C, 15 min). Further, after microwaving (900 W, 2450 Hz, 1.5 min), weight reduction was 11.9% as a result of a more efficient moisture removal from the vegetable tissues [30].

Besides, it is well known that *sous-vide* treatment causes changes in the color of vegetables. For instance, L* value of asparagus spears decreased from 54.1 to 42.6, making them darker after the processing [23]. Furthermore, this value was even lower than with boiled asparagus (44.4). A reduction in hues parameter from 115.8 (raw) to 112.8 (*sous-vide* samples) was noticed due to conversion from green to olive-green h°. This was a consequence of chlorophyll transforming to pheophytin [31], which was also lower than the value obtained for asparagus after boiling (113.9). In the case of broccoli florets, a 60% increase in a* value was observed after *sous-vide* treatment (90 °C, 15 min), while boiling (100 °C, 3.5 min) reduced the value of this parameter by 36% [28]. Moreover, boiling did not modify h° parameter when compared to the raw florets and stems but *sous-vide* did reduce this value by 13% and 19%, respectively. dos Reis et al. [29] analyzed color parameters in organic cauliflower inflorescences after boiling (100 °C, 5 min), steaming (final temperature 95 °C), microwaving (800 W, 4 min) and *sous-vide* processing (90 °C, 20 min). In all cases, an L* parameter reduction was observed. As regards parameter a*, *sous-vide* cauliflower registered lower values compared to the other cooking methods. 

Carrot color is attributed to the presence of carotenoid pigments and a* value correlates well with sensory acceptability. Patras et al. [32] reported that when compared to a fresh sample, carrot slices prepared via *sous-vide* (90 ° C, 10 min) decreased 12% in this parameter, while for samples boiled in water (until core temperature was 70 °C), the reduction was 30%. Moreover, the authors also observed that the losses of color red continued during storage. They reported a decrease of 27% for *sous-vide* carrot disks and a reduction of 32% for boiled samples after 20 and 5 days of storage, respectively. Consequently, it was concluded that *sous-vide* carrots were superior in color as compared to water-immersed cooked samples. 

Trejo Araya et al. [33] compared the appearance of *sous-vide* carrot sticks (90 °C, 5 min in contact with water) to raw samples. Intensity of orange, visual firmness, brightness, surface moisture and flexibility (judged using finger touch) were used as indices. Panelists assigned higher scores to *sous-vide* carrots in all categories except for visual firmness. Concerning perception of texture, the processed vegetables were classified as more fibrous than raw samples but not significantly different. Furthermore, release of higher levels of moisture were perceived in the mouth. For instance, when compared to raw carrots the crunchiness and chewing time of *sous-vide* samples decreased because of water release during mastication.

Relating to textural aspects of broccoli, *sous-vide* samples (90 °C, 15 min) registered less stem softening than boiled ones (100 °C, 3.5 min). Shear force values decreased by 49.0% for the former and 65.7% for the latter, as compared to the initial stem firmness (36.5 N). It was speculated that when lower temperature and vacuum packaging were applied, cell wall disruption was minimized, and stem firmness was less affected [28]. Similarly, dos Reis et al. [29] measured values of 72.6 N for fresh organic broccoli, 16.8 N for broccoli submitted to *sous-vide* processing (90 °C, 20 min) and 12.7 N for broccoli subjected to conventional boiling (100 °C, 5 min). In contrast, for cauliflower, the same authors reported that firmness was less affected by boiling (33.5 N) than by *sous-vide* (15.6 N). The lowest shear force exhibited by *sous-vide* inflorescences could be associated with 3.2% water losses measured for these samples, against an increment of moisture by 1.0% for boiled samples.

Regarding aroma, studies using broccoli florets, green beans and carrots cooked via *sous-vide*, authors stated that these samples retained more aromatic volatile components than boiled samples [20,34,35]. Moreover, Rinaldi et al. [36] reported that *sous-vide* Brussel sprouts and carrots had different volatile profiles as compared to the steamed samples. This could be attributed to reduced degradation due to the lower presence of oxygen because of the vacuuming. 

In conclusion, findings generally agree that *sous-vide* vegetables retain more aroma and taste than conventional cooked samples. Certainly, preparation via *sous-vide* avoids leaking of hydrophilic components into boiling water and these substances are related to the perceived flavor by consumers. Finally, to the best of our knowledge, no data are available regarding the effects of *sous-vide* on fruits’ quality. Even though fruits are usually eaten raw, some varieties can be cooked. For instance, apples and pears could be submitted to *sous-vide* treatment to potentiate flavor and promote consumer acceptance, hence in this sense more studies are required.

### 2.2. Changes in Nutrients and Phytochemicals of Sous-Vide Vegetables

There are very few available reports about the impact of *sous-vide* on vegetables on the bioactive compounds such as carotenoids, phenolic compounds, vitamin C and/or their antioxidant capacity. Chiavaro et al. [37] studied the changes in the phytochemical contents and antioxidant capacity of carrot slices and Brussel sprouts after *sous-vide* processing (100 °C, 20 min) and refrigerated storage at 4 °C for 1, 5 and 10 days, with a reheated final step for 20 min in a water bath at 60 °C. Authors compared the results of *sous-vide* processing/refrigerated/reheated products with the corresponding raw and oven-steamed products. In this regard, *sous-vide* carrots and Brussel sprouts at day 1 of storage at 4 °C showed higher contents of carotenoids in comparison to raw and steamed products. For instance, *sous-vide* carrots exhibited carotenoids content 1.8 and 1.1 times higher than raw carrots and those prepared by steaming. This increase was particularly evident for α- and ß- carotenes, and it was probably due to the reheating of the *sous-vide* processing that may more efficiently release carotenes that normally reside in cellular crystals and are bound by the complexes with protein and/or residual membranes [37,38]. Moreover, carotenoids were released from both, carrots and Brussel sprouts, during storage beyond day 1. Here, a significant increase was observed in *sous-vide* samples as compared to the raw and steamed carrots (total carotenoid content in *sous-vide* carrots at day 10 of storage was 2.3 and 1.2 times higher than the total carotenoids in a raw and steamed carrots, respectively). Similarly, *sous-vide* carrot slices (90 °C, 10 min) preserved the total carotenoid content better than conventional cooking (samples were boiled in water until core temperature reached 70 °C) [32]. Carotenoids are lipophilic compounds are less prone to leakage during *sous-vide* processing, however they are still sensitive to oxidation. Thus, *sous-vide* vegetables are more protected against oxidative carotenoid degradation, not only during the cooking, but also throughout the refrigerated storage, as their contact with oxygen is limited due to vacuum-packaging. 

Phenolic compounds seem to be better preserved in the vegetable products during *sous-vide* as compared to conventional cooking methods, e.g., boiling or steaming. Similar to carotenoids, this was probably due to limiting oxidation of these compounds under vacuum conditions [37]. In that sense, Martínez-Hernández et al. found a slight increase (lower than 1.4 times) in the phenolic compounds of kailan-hybrid broccoli cooked by *sous-vide* (90 °C, 15 min) as compared to the raw produce [28]. Moreover, Štěrbová et al. [39] reported a gain of 5% in the total phenolic content of Sacha inchi kernels cooked by *sous-vide* at 100 °C for 135 min. Additionally, Chiavaro et al. stated that *sous-vide* better preserved phenolics in carrots slices with slight increase in their content (of less than 5% as compared to the raw samples). This was particularly emphasized for some flavonoids (quercetin, kaempferol and luteolin) and hydroxycinnamic acid derivates, such as caffeic and ferulic acids [37]. On the other hand, Baardseth et al. [40] reported that *sous-vide* (100 °C, 15 min) of blanched/frozen green beans had no effect on the concentration of total phenolics. It was concluded that *sous-vide* or other types of cooking do not increase the content of phenolic compounds in vegetables, rather they facilitate the extraction by increasing the yield in the extracts (not in the vegetables). 

However, the high water solubility of phenolic compounds and their thermal instability (of some of them) with the higher temperatures and the negative pressures of vacuum may cause, during *sous-vide,* forced lixiviation to the extra-cellular media and/or their possible thermal degradation. In this way, the phenolic compounds of inflorescences and co-products of several Brassica vegetables were significantly reduced with *sous-vide* (*T =* 80 °C, t = 15 min for inflorescences and leaves, and T = 80 °C, t = 90 min for stems) in relation to raw products [41,42]. Furthermore, Patras et al. [32] reported a decrease of 29.2% of the total phenolic content in carrot slices that were cooked by *sous-vide*. This was in addition to Chiavaro et al. [37] who found that *sous-vide* in Brussel sprouts decreased phenolics by 14% in comparison to the raw samples.

Vitamin C includes ascorbic acid and its oxidation product, dehydroascorbic acid [43]. It was believed that both of these compounds have several biological activities that include cancer-protective capacities in the body. The content of vitamin C in fruits and vegetables can be significantly reduced during processing and storage due to its solubility in water, thermal sensitivity and proneness to oxidation. Thus, it is expected that vegetables cooked by *sous-vide* may have decreased vitamin C content, but still at lower levels than conventional cooking. In a way, this effect is similar to other bioactive compounds, i.e., as the absence or the very low presence of oxygen in the pouches may mitigate ascorbic acid oxidation. So it is somewhat expected that thermal processing by *sous-vide* significantly reduced the vitamin C content of Brassica vegetables and co-products [41]. For instance, *sous-vide* of inflorescence and stem of Broccoli *cv.* ‘Parthenon’ experienced vitamin C degradation near to 84% and 67%, respectively. However, authors observed that the reduction was significantly higher after steaming when compared to *sous-vide* for some Brassica vegetables. This was probably due to the reduced amount of oxygen present when cooking by *sous-vide* [41,42]. On the other hand, good retention of vitamin C was observed for all *sous-vide* carrot slices with a significant slight reduction at the end of procedure (at day 10 of storage at 4 °C) of about 6%. Moreover, *sous-vide* of blanched/frozen green beans slightly decreased the levels of ascorbic acid, while conventional cooking by boiled water induced a loss of vitamin C close to 50% [40]. Therefore, *sous-vide* processing has potential to preserve vitamin C in vegetable foods. Probably, the percentage of retention depends on quality of vacuuming in the package and harshness of thermal treatment. 

Lastly, some authors studied the effects of *sous-vide* on the antioxidant capacity content of several vegetable products. *Sous-vide* resulted in a significant reduction in the antioxidant potential trough the FRAP (Ferric Reducing Antioxidant Power) assay of all studied inflorescence and leaves of Brassica vegetables. Moreover, for some parts of some varieties, including the inflorescences of broccoli cv. ‘Marathon’ and broccoli cv. ‘Parthenon’, *sous-vide* resulted in losses that ranged from 40% to 50% of antioxidant potential as compared to the raw products. However, the antioxidant activity of the samples measured by the DPPH assay (2,2,1-diphenyl-1-picrylhydrazyl radical scavenging) increased after *sous-vide*. For instance, this increase in antioxidant activity was higher after the *sous-vide* processing of the inflorescences of broccoli cv. ‘Marathon’ (4 times higher than the raw product) and the stems and inflorescences of broccoli cv. ‘Pastoret’ (5 and 1.3 times higher than raw products). The increase in the antioxidant activity could be caused by the release of insoluble antioxidants or by the formation of new ones from temperature-dependent reactions, and/or due to water loss during processing [41,44]. It is possible that the differences between the antioxidant activities obtained using FRAP and DPPH might be due to the different principles on which these methods are based: acceptance of hydrogen atoms and electrons from antioxidants for DPPH and FRAP assays, respectively [45]. In agreement, Martínez-Hernández et al. [28] reported that cooking kailan-hybrid broccoli by *sous-vide* increased between 4.7- to 5.4-folds of the initial total antioxidant capacity as compared to uncooked samples. Moreover, *sous-vide* induced a great increase of the total antioxidant activity in carrot slices and Brussel sprouts by the great enhancement of carotenoids and flavonoids (only for carrots), as well as the marked retention of ascorbic acid shown by these samples [37]. 

In conclusion, *sous-vide* vegetables had higher amounts of bioactive compounds (e.g., carotenoids, phenolics and ascorbic acid) than conventionally cooked alternatives (e.g., boiled or steamed). In addition to this, *sous-vide* vegetables retained higher levels of antioxidant activity. Moreover, high temperature also induced modifications in the vegetable matrix than can be positive for the release of bioactive compounds. The *sous-vide* packaging provides protection from oxidation, and hence provides consumers a vegetable with richer phytochemical content. Lastly, further investigations are needed to evaluate if changes in the vegetable matrix are due to *sous-vide* processing, in other words, to clarify if this type of processing provides phytochemicals that are more easily released from the matrix, absorbed in the gastrointestinal tract (bioaccessibility), and available for physiological processes (bioavailability).

### 2.3. Microbiological Concerns of Sous-Vide Vegetable Products

The safety concerns of *sous-vide* products, particularly concerning spore-pathogen bacteria, needs to be carefully evaluated on a product-by-product basis. The maximum growth temperature for many pathogenic microorganisms growing on food products is between 42 and 49 °C, and some of them have been observed to grow slowly at temperatures between 50 and 55 °C. Therefore, the temperatures being used for *sous-vide* cooking might be close to, or overlap, the growth temperature ranges of foodborne pathogens [46].

The most often contaminants found in vegetables include *E. coli*, *Enterobacter* spp., *Klebsiella* spp., *Salmonella typhi*, *Serratia* spp., *Providencia* spp., *Staphylococcus aureus* and *Pseudomonas aeruginosa,* and other potentially pathogenic microbes [47]. In addition to these, some other types of vegetables are more susceptible to spoilage by other types of microorganisms, like *Bacillus cereus*, *Campylobacter jejuni*, *Clostridium botulinum*, *E. coli O157: H7*, *L. monocytogenes*, *Salmonella* spp., *Shigella*, *Staphylococcus* and *Vibrio cholera* [48]. Most of them are facultative anaerobes, which means that the cells are able to survive and grow in an environment with or without oxygen. Table 1 summarizes selected foodborne bacteria with possible growth and toxin production in temperature ranges below 34 °C; thus, the temperature range is shown to indicate the upper limits of temperature where growth has been observed to occur.

Slow heating of the product to the cook temperature may provoke a heat shock response by the microbes, making them more heat-tolerant to the cooking temperature. Therefore, good operating practice is oriented toward pre-heating the water bath to the appropriate cook temperature. This is especially important when the cook temperature is close to the upper growth temperature of a given microb as this may result in a decrease in the rate of its inactivation. At temperatures below 55 °C, spore-forming bacteria may survive and promote germination, resulting with an increase in bacterial cell number during the cooking, and consequently, increase the incidence of foodborne illness. To date, there is little to no scientific evidence to support any prediction model for foodborne pathogens’ inactivation in vegetables and/or seafood at cooking temperatures at or below 55 °C, therefore, the safety of *sous-vide* cooked at temperatures below 55 °C cannot be assured. An alternative could be to add bio-preservatives (nisin and organic acids) additionally to apply non-thermal hurdles by using some innovative non-thermal technologies or to use time-temperature indicators in the packages for recording the storage history of a product [49,50].

A recent line of investigation [51] introduced the use of rosemary essential oil (REO) as a natural antibacterial and antifungal to process fresh-cut potatoes. Firstly, this essential oil would allow avoiding the use of synthetic preservatives due to the antimicrobial activity related to the presence of components such as 1,8-cineole, α-pinene, borneol, ver-benone, and camphor [52]. Secondly, the combination of REO with the characteristic aroma of vegetables could potentiate the final product flavor. The results showed that the synergistic use of REO and vacuum packaging, combined with refrigerated storage, controlled the growth of mesophilic bacteria and *Enterobacteriaceae* in minimally processed potatoes destined for cooking with the *sous-vide* method after 11 days of storage. 

Sebastiá et al. [53] evaluated the microbiological quality of broccoli, courgette, potatoes and carrots processed via the *sous-vide* method (100 °C, 15–20 min, except for courgette, which was heated for 5 min), chilled below 3 °C and stored (0, 15 and 30 days). Broccoli had the highest aerobic plate counts in all the storage periods. Authors attributed these results to the inherent morphology of broccoli. Broccoli inflourescences have hydrophobic pockets, which develop isolated areas that are not reached in the washing process. Therefore, it is necessary to reduce the organic material present in the samples with chlorine and improve the disinfection stage [54,55,56].

Neither enterotoxigenic staphylococci nor staphylococcal enterotoxins were detected in any of the four *sous-vide* cook–chill preserved vegetables. Detection of both staphylococci and staphylococcal enterotoxins were done because of the several coagulase-negative staphylococci also detected as enterotoxins [57,58]. Moreover, the presence of coagulase-negative staphylococci as *S. epidermidis*, a normal skin commensal, in foods reflects poor hygiene. Both enterotoxigenic staphylococci and their toxins can be used as indicators to assess the risks of vegetable contamination by staphylococci [58,59]. Additionally, there are bacteria that grow at low temperatures and survive mild heat treatments, e.g., *L. monocytogenes* and *E. coli* [60,61]. However, neither of these species were detected in the studied vegetables. Authors asserted that the absence of these microorganisms in the samples is mainly due to the microbial quality of raw vegetables combined with strict temperature control during the process. This is in agreement with the Sous-Vide Advisory Committee (SVAC, 1991) [62] considerations that indicated that microbiological safety depends on thermal process intensity, cooling speed, the final temperature, temperature monitoring and time of refrigerated storage. Moreover, it is important to check the packaging integrity throughout the entire process to assure storage and safety [53].

Rinaldi et al. [63] carried out microbiological analyses (aerobic and anaerobic total plate counts, mesophilic lactic acid bacteria, yeasts and molds) of steamed and *sous-vide* carrots and Brussel sprouts (20 min under steam at 100 °C) after 1, 5 and 10 days of refrigerated storage. Both group of carrots showed microbiological counts that were always lower than 1-log colony-forming units per gram (CFU/g), and even after 10 days of storage at 4 °C. Therefore, both thermal treatments appeared efficiently to diminish the initial counts. Similarly, Sebastiá et al. [53] reported aerobic total plate counts of <1 log CFU/g for carrots submitted to the *sous-vide* process for up to 30 days under refrigerated storage. In the case of Brussel sprouts, both steamed and *sous-vide* samples showed a decrease of all microbiological counts. The values of aerobic total plate counts were 3.46 log CFU/g for steamed samples and 2.34–3.15 log CFU/g for *sous-vide* sprouts. Moreover, *sous-vide* Brussel sprouts exhibited all other microbiological counts (<1 log CFU/g) lower than the values observed for steamed samples for up to 10 days of refrigerated storage. Authors also associated these results with an appropriate time–temperature combination, particularly, controlled heating/chilling steps that reduced initial flora more efficiently than steaming [53]. 

The table below illustrates the recent literature review on the influence of sous-vide cooking on the quality parameters of vegetables (Table 2).

## 3. *Sous-Vide* Applications in Seafood Processing 

The demand for natural and lightly processed convenient seafood products is constantly growing, therefore the efforts in the processing of seafood and fish that will ensure safe and high-quality products resulted in the development of alternative process technologies such minimal cooking techniques [68]. Marine organisms have been recognized as a valuable dietary source of high quality bioactive components such as long-chain omega-3 fatty acids (PUFAs), easily digestible proteins, non-protein nitrogen compounds, fiber, taurine, sterol, and pigments. They also contain unique components that are not present in terrestrial organisms [69]. However, because marine organisms muscle lipids are highly prone to oxidation due to their high content of polyunsaturated fatty acids, their sensory and nutritional quality can be rapidly destroyed without suitable handling and processing, leading to rancidity and development of off-flavors. The most common methods that are used for the vegetable processing (e.g. stewing, microwaving, roasting, boiling and steaming) [70] could be used in seafood processing too [71,72,73]. Nevertheless, cooking facilitates several undesirable physicochemical reactions, among which, lipid oxidation is with the most detrimental consequences on cell membranes and denaturation of heme-proteins [74]. During cooking of vegetables, meat or seafood, water-soluble nutrients e.g., vitamins, minerals are typically lost at higher temperatures through evaporation and as exudates leave the food. This includes bioactive compounds and antioxidants, which are essential for maintaining a healthy immune system [75]. Some water-soluble proteins also may be lost with the water during cooking process. Higher cooking temperatures lead to myofibrillar protein shrinkage while decreased binding force between proteins and water that results with the decreased water holding capacity (WHC) in the myofibrils [76]. Sous-vide cooking could better preserve the stability of a secondary structure of proteins as compared to classical cooking procedures. Accordingly, Wan et al. [76] concluded that sous-vide cooking could be used as healthy alternative as it was proved to be helpful in maintaining the quality of largemouth bass fillets. However, in order to preserve sensory and nutritional quality during thermal processing of marine organisms, careful observation of operating technological parameters is required.

It was established that both temperature and cooking time have an effect on lipid oxidation in seafood products [7]. Furthermore, higher temperatures induce various biochemical reactions, protein aggregations and conformations, which change the tissue gaps in fish muscles [76]. Głuchowski et al. [77] by using higher process parameters in the sous-vide cooking of Atlantic Salmon (Salmo salar) achieved a similar intensity of cooked fish odor and flavor without significant degradation in the texture. However, vacuum pretreatment can be utilized to isolate oxygen thus avoid biochemical reactions that require oxygen, but also to minimize the reduction of lipids damage during heating process [76]. 

Dominguez-Hernandez et al. [10] confirmed that low-temperature over long-time cooking (LTLT) of meat offers multiple advantages over traditional high temperature cooking [6], such as reduced heat degradation of proteins and lipids and lower loss of liquid nutrients [78]. In a line with this, sous-vide might be useful even for processing of marine organisms like cephalopod or fish and seafood maintaining their fresh-like characteristics as with the produce [79]. However, there is a scarcity of literature concerning the sous-vide applications in seafood processing.

Mouritsen and Styrbæk [80] recently reviewed the future perspective of novel trends in cephalopod processing with respect to gastronomy. In this case increased demand for slightly processed seafood with a prolonged shelf life led to the application of sous-vide in order to obtain high quality seafood products [81]. Nevertheless, sous-vide has shown some limitations in seafood processing. When considering vacuuming, its lower degree implies that the higher pressure is applied, and high pressure is not recommended for sous-vide processing of fish fillets as the texture of fish is very delicate and gentle, and high pressure might initiate unwanted damages of the tissue. Consequently, such type of food cannot be completely vacuumed and the residual pressure inside the package is typically around 100–120 mbar, that reflects the difficulties for sous-vide processing [16]. Non-desirable reactions during sous-vide cooking like degradation of vaccum-seal bag, might also take place. Seafood contamination can easily occur by migration of plastic derived compounds into product during sous-vide cooking [82].

Cooking and preparing squid and squid or octopus by sous-vide technology are one of the biggest challenges today, as both of them are not always of uniform quality [83]. Like the abalone and clam, squid and octopus must be cooked very slowly to prevent the muscle fibers from toughening. Moderate temperature during sous-vide cooking of cephalopods retains its structure. Cuttlefish is quite difficult to cook, because it gets tough or rubbery, therefore must be tenderized before cooking. Cooking with sous-vide technology is beneficial as the low temperatures prevent the meat from contracting and gives a soft, tender food while plastic foil and vacuum prevents the loss of aromatic compounds.

In order to preserve the quality and safety of seafood sous-vide products, combinations with other processing steps such as different packaging technologies are often required. There is a possibility for sous-vide cooked marine organisms to be stored at modified atmosphere packaging (MAP) to achieve prolonged shelf life combined with low storage temperatures [84]. Lightly processed salmon (Salmo salar L.) by sous-vide cooking (45 °C for 15 min, 55 °C for 18 min, 65 °C for 21 min) was stored under modified atmosphere (MA) (60% CO_2_ balanced with N_2_) and soluble gas stabilization (SGS) (100% CO_2_) at 4 °C for up to 24 days [85]. Authors found that SGS significantly improved shelf life of processed salmon by prolonging the lag-phase and slowing the growth rate of naturally occurring and inoculated bacteria. This study confirms that sous-vide processing at lower temperatures could provide inhibition of the bacterial growth, while improving chemical quality compared to traditional processing.

### 3.1. Changes in Physicochemical Properties and Sensory Quality of Sous-Vide Seafood Products

Recently, sous-vide has been considered as a potential technique that could have an impact on the improvement of taste. In this regard, the intensification of umami taste by producing more glutamate as a result of tenderizing meat upon the sous-vide cooking at 54 ℃ or 64 ℃ during 0.5, 1, 1.5, 2, 3, 4, 6, and 8 hours has been explored [86]. However, authors concluded that aside from texture analysis and amino acid composition, full chemical analysis with determination of free nucleotides combined with a sensory evaluation could be useful approach to support this hypothesis. Another research suggested that the preparation of fermented fish products, such as fish sauces, could be used to provide free glutamate and inosinate. Fresh fish such as mackerel, anchovies and sardines are a particularly good source of adenosine triphosphate (ATP) that can be enzymatically turned into inosinate, soon after the fish is captured, and then fermented to create large quantities of glutamate [87]. 

In contrast to fish, cephalopods can be completely vacuumed inside the package, prior to sous-vide thermal treatment, because, this organisms have strong muscular tissue and firmness. The firmness is probably associated with changes on connective tissue structure, which lies immediately below the skin and is reported to be resistant to processes of the autolysis [88,89]. Muscle firmness is closely related to collagen content in seafood thus the squids are especially difficult to cook. Most methods require either quick frying or poor and slow roasting which can still result in hardening of the hard chewing food. Mouritsen and Styrbæk [80] reported that the mantle from cephalopods, like Sepia officinalis and Loligo forbesii, need very short heating at low temperatures (50–60 °C) to become more tender and ensure succulent structure. Low temperatures in sous-vide cooking of squids may ensures a soft and melt-in-the-mouth texture. On the other side, increased temperatures during sous-vide cooking enhance protein denaturation and coagulation of sarcoplasmic proteins on the surface and significantly alter the color. Moreover, protein denaturation plays a major role in the toughening of the texture of muscle products [85].

Cooking methods were also shown to affect the pH values. For instance, the pH of the largemouth bass increased during *sous-vide* cooking [76] due to the formation of disulfide bonds during the cooking process [90]. Croptova et al. [7] found increased pH values for *sous-vide* cooked mackerel samples at 60 °C for 10 min, while lower pH values were recorded in *sous-vide* cooked samples at 75 °C and 90 °C for 10 min on the seventh day of chilled storage. Authors supported previous explanation of this trend by the generation of trimethylamine and total volatile base nitrogen from either microbial or endogenous enzymatic degradation [91]. In addition, cooking losses were found to be significantly affected by *sous-vide* operating conditions and chilled storage. Especially, higher temperatures of *sous-vide* cooking enhanced cooking losses in mackerel samples, consequently negatively influencing the product quality. On contrary, the length of chilled storage had a positive contribution to the retention of water in the fish, probably due to structural changes occurring in protein matrix of the fish muscles and connective tissues after cooking [92], including reabsorption of water released by unfolded myofibrillar proteins and its distribution between intracellular and extracellular compartments [93].

Color changes that may occur during cooking are mainly attributed to protein denaturation that is reflected in meat by a more white color during the cooking process [94]. Both instrumental color parameters, lightness (L*) and yellowness (b*), revealed a significant increase throughout chilled storage of *sous-vide* as compared to the color parameters of the raw mackerel fillets [7]. Increased temperature of *sous-vide* significantly affected increased L* value. This is possible due to higher denaturation and aggregation of sarcoplasmic and myofibrillar proteins that reflects in increased light scattering [95]. Wan et al. [76] observed significant increase of whiteness in *sous-vide* cooked largemouth bass samples as compared with the fresh samples. Moreover, in the same study, authors concluded that the texture attribute of elasticity was greater pronounced in the cooked samples than in the raw ones, but in other indicators (hardness, chewiness and resilience) results were opposite. The decreasing of hardness and chewiness in cooked fish may be attributed to the lipid oxidation, which can produce numerous products (mainly aldehydes) that might cause the myofibrillar protein cross-linkages, thus leading to structural changes in proteins. 

Regarding textural changes occurred during *sous-vide* cooking, recent study demonstrated a significantly lower values of breaking strength during chilled storage in comparison to the fresh samples of mackerel fillets. Interestingly, the fillets’ firmness increased in proportion with raised heating temperature (60 ℃ *vs*. 90 ℃), that may be explained by the heat-induced hardening of the fish muscles after denaturation of myofibrillar and sarcoplasmic proteins. Water losses from the muscle tissue were more intensive at higher temperature (90°C), so contributing to mackerel samples toughening [7]. 

Humaid et al. [81] investigated the use of high pressure processing (HPP) at 150 and 350 MPa for 5 and 10 min for *sous-vide* cooked lobster tails (*Homarus americanus*) at 65 °C/10 min. Results showed that the use of moderate pressures significantly influenced the texture and color of lobster tails, whereas processing time had a milder effect. HPP at 350 MPa significantly increased L* values of *sous-vide* cooked lobster tails, but without considerable impacts on the overall acceptability by a consumer panel. Another study of Humaid et al. [96] established the effectiveness of HPP in extending refrigerated shelf life of vacuum-packaged raw lobster tails while HPP pretreatment did not positively contribute to additional shelf life extension for *sous-vide* samples. Finally, the authors concluded that the use of HPP to vacuum-packaged lobsters for subsequent *sous-vide* cooking has a potential in the development of novel ready-to-eat functional seafood products able to be stable during refrigerated storage. 

### 3.2. Changes in Nutrients and Phytochemicals of Sous-Vide Seafood Products

Next to the temperature/time regimes with sous-vide, the occurrence and the extent of the reactions related to changes of nutritive quality will depend on the heat transfer medium (liquid water or steam) and from exposure to oxygen (meat vacuum packed or not). Numerous reactions could occur during the cooking, affecting lipids and the volatile profile of seafood such as lipid hydrolysis, lipid oxidation and degradation of *nitrogenous* compounds (proteins, amino acids and trimethylamine N-oxide, TMAO) via Maillard reactions or other types of deteriorative reactions [82].

Protein denaturation is a major event in the cooking of meat or seafood, which is less pronounced in sous-vide. Hence food structure is somewhat better retained along with color and taste which can be very attractive for consumers [97]. Plastic foil prevents the loss of aromatic volatile compounds and water during the sous-vide, which contributes to juiciness and tenderness of meat and enhanced sensory attributes [16]. Roldán et al. [98] reported the formation of volatile compounds in the amino acid-involved reactions during sous-vide processing (60 °C for 6 and 24 h, 80 °C for 6 h) of lamb meat. As suggested, the aromatic volatiles were associated with a specific stronger meaty flavor and roast notes, due to which fewer spices and less salt was required. 

A healthy diet (lower salt diet) may help prevent certain long-term (chronic) diseases thus, it is of great interest to find the right balance between these different nutrients to achieve maximum health benefits. Djordjević et al. [99] revealed that fish as a food, in addition to the valuable content of proteins, minerals and vitamins, is particularly attractive to the consumers as it represents a very rich source of essential fatty acids, which play a role in the prevention of many human diseases [100]. 

Marine organisms have noticeable content of valued fatty acids and high temperature treatments may negatively affect the stability of these essential nutrients. Except for octopus, cephalopod is considered as a rich source of unsaturated fatty acids, in particular unsaturated omega-3 docosahexaenoic acid (DHA) and eicosapentaenoic acid (EPA) [101]. Therefore, cephalopods comprise great source of lean low-fat protein food and knowledge about the physical structure of the cephalopod’s muscles (squid, octopus and cuttlefish) can be a useful guide for gastronomy and nutrition.

A recent study evaluated the impact of mild culinary treatments such as boiling (100 °C, 10 min), steaming (100 °C, 10 min), and *sous-vide* cooking (85 °C, 20 min), on the lipid composition and volatile components of farmed and wild European sea bass (*Dicentrarchus labrax*) [82]. Obtained results showed that the mild oxidative conditions of the three performed cooking methods did not provoke the hydrolysis of sea bass triglycerides, phospholipids or retinyl esters. In addition, no significant lipid oxidation took place during cooking. However, a slight oxidation of unsaturated acyl groups during cooking has been noticed, yielding several volatile secondary oxidation compounds of low molecular weight. Steaming and *sous-vide* cooking, in contrast to boiling, resulted in a slight oxidation of unsaturated acyl groups, leading to the formation of alcohols, aldehydes, ketones, alkyl furans and acids, that consequently positively affected aromatic profile. Volatile profiles of cooked samples were also enriched by degradation of nitrogenated fish components (includes Maillard-type reactions) that was considered as acceptable from a sensory point of view.

### 3.3. Microbiological Concerns of Sous-Vide Seafood Product

Microbiological deterioration in perishable products such as seafood occurs rapidly due to neutral pH, high water activity, and nutritional composition. When observing seafood safety of sous-vide seafood products, pathogenic bacteria that must be considered are classified into three groups. Namely: (i) bacteria naturally present in the habitat of the consumed species, such as Vibrio spp., non-proteolytic Clostridium botulinum type B, E and T, Plesiomonas shigelloides and Aeromonas spp; (ii) bacteria present in the environment in general (Listeria monocytogenes, proteolytic Clostridium botulinum type A and B, Clostridium perfringens and Bacillus spp; and iii) bacteria which have their usual habitat in man or animals (Salmonella spp., Shigella spp., Escherichia coli, Campylobacter jejuni and Staphylococcus aureus) [102]. The presence of bacteria group (i) and (ii) in live or fresh raw fish is not a common safety concern because tissue concentrations are too low to produce disease. Heat is lethal to microorganisms, but each species has its own particular heat tolerance, and there are many factors affecting their thermal resistance. The process is dependent both on the exposure time and on temperature required to achieve the desired death rate. Therefore, it is essential to determine the thermal death kinetics (D and z-values) of target bacteria in different food substrates and to characterize the time durations to be applied at certain temperatures according to this data. The decimal reduction time (D value) is the time in minutes at a given temperature required to destroy 1 log cycle (90 %) of the target microorganism. The z-value reflects the degrees of temperature change necessary to change the D-value by a factor of 10 [103]. For example, time (minutes) sufficient to achieve a pathogenic load reduction of six orders of magnitude, or a 6D reduction (six logarithms, e.g., from 10^3^ to 10^−3^) of Listeria monocytogenes for meat, fish, or poultry in water baths from 60 °C to 66 °C based on 2 min of cooking at 70 °C with z = 7.5°C is given in Table 3. 

However, the accumulation of large numbers of pathogens (Vibrio spp.) in filter-feeding shellfish represents a risk, especially since shellfish are commonly eaten raw. Pre-harvest contamination with pathogens from the group (iii) may present a risk since in some cases a very low infective dosage is sufficient to cause serious disease [102]. Predictive models for thermal inactivation and growth of microbes under sous-vide conditions were recently reviewed. Here some of the limitations of current modelling approaches were also observed, particularly for a longer processing at lower temperatures [8]. Though regular cooking procedures will eliminate the risks of contamination, advanced knowledge is required regarding their origin, biology, physiology, ecology, survival, growth and prevalence in seafood and related products, along with the epidemiology and symptomatology of the diseases with which they are associated [105].

Fish or seafood sous-vide processing requires lower cooking temperatures (50–75°C) with cooking time for several hours or even days. Sous-vide cooking is considered relatively safe for fish and seafood due to the fact that the food is consumed immediately after preparation, usually with a delay no longer than two hours, during which it is stored at temperatures above 54.4°C to prevent or slow down the reproduction of pathogenic bacteria [16]. The European Union guidelines recommend that the minimum heat treatment for sous-vide pasteurization should be equivalent to heating at 70 °C which should be reached throughout 2 minutes [15]. Picouet et al. [106] for sous-vide cooked salmon loins established 4.5 log CFU/g for TVC (Total viable aerobic count) and 3.0 log CFU/g for Enterobacteriaceae under at 40.7 ± 0.1 °C for 19 min of cooking conditions. Such a successful sous-vide cooking at a lower temperature was attributed to the influence of additional high pressure processing (210, 310 and 400 MPa for 5 min at 10 °C).

However, insufficient heat treatment is the major problem in sous-vide processes applied at low temperatures. Therefore, sous-vide cooking has been lately combined with the use of natural antioxidants to improve the efficiency of cooking process in terms of food safety during storage [107,108]. This is in compliance with the latest trends in the food industry where different processing procedures could be combined to promote the process efficiency [50]. Also, there is an increased awareness towards replacing the use of synthetic antioxidants in food processing by the addition of natural ones [109], accordingly sous-vide cooking supplemented by the addition of natural antioxidants could be perspective tool for foods preservation. Several studies have been shown this concept as a promising alternative in preserving quality of fish products. Alves et al. [110] among oregano, basil and rosemary extract confirmed oregano as the most effective for preservation of sous-vide cooked tilapia fillets. Though the addition of oregano essential oil (EO) lowered the pH of sous-vide cooked salmon, which may favor the microbial inactivation, Dogruyol et al. [108] found that L. monocytogenes was more rapidly inactivated due to antibacterial effect of different compounds present in EO, like carvacrol and thymol [108]. 

Limiting storage times is another way to control the growth of pathogens in sous-vide seafood. Table 4 provides the key findings regarding the impact of shelf life on the quality parameters of sous-vide seafood products. Here, storage for extended periods of time is not recommended unless the product is frozen [111]. Cooled sous-vide seafood products should be stored in cold holding units and maintained at an internal temperature of 3 °C or lower to prevent growth of anaerobic spore-forming pathogens such as non proteolytic Clostridium botulinum, Clostridium perfringens and Bacillus cereus. If the food has a pH > 4.6 or available water ≥0.92, cold holding times should be limited, whereas Baldwin [6] recommends that such sous-vide products can be kept for maximum of 30 days.

Table 5 summarizes the recent literature review on the influence of *sous-vide* cooking on the quality parameters of seafood.

## 4. Conclusions

*Sous-vide* cooking is becoming increasingly popular as a convenient and reliable method to produce healthy and high-quality vegetable and seafood products in the home, food service environment or food industry. Lately, due to the lack of time available for the consumption and preparation of meals and foods, *sous-vide* is gaining in popularity among consumers as an advantageous approach over conventional thermal treatments. *Sous-vide* cooking employs much lower temperatures than traditional cooking, and therefore it is possible to obtain more nutritious food products with well-retained bioactive compounds, which has significance from the health perspective. Moreover, studies have shown that *sous-vide* cooking could provide foods with higher nutritive value and more pronounced color characteristics, texture properties and sensory attributes than corresponding raw untreated food. Currently, marine organisms are much more in demand than vegetables for *sous-vide* processing due to higher risk of spoilage during shelf life. Therefore, major concerns related to *sous-vide* processing involve the microbiological safety of the products. In order to improve food safety, recent research combines *sous-vide* technology with natural antioxidants or innovative (non)thermal technologies that resulted in beneficial effects for both quality and safety issues. In conclusion, *sous-vide* has a great potential for future applications and to achieve safe foods with improved sensory and nutritive characteristics.

## Figures and Tables

**Table 1:** Temperature range (above 33 °C) of possible growth and toxin production for selected foodborne bacteria.

Bacteria		Temperature (° C)																					
		34	35	36	37	38	39	40	41	42	43	44	45	46	47	48	49	51	52	53	54	55	56
*Bacillus cereus*	G																						
	T																						
*Campylobacter*	G																						
*Clostridium botulinum*	G																						
	T																						
*Clostridium perfringens*	G																						
*Listeria monocytogenes*	G																						
*Salmonella spp.*	G																						
*Shigella*	G																						
*Staphylococcus aureus*	G																						
	T																						
STEC	G																						
*Yersinia*	G																						
*Vibrio parahaemolyticus*	G																						

* STEC-shiga toxin-producing *E. coli*. G—growth 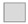; Optimun for growth 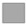; T—toxin production in food 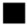.

**Table 2:** Literature review on quality indices of *sous-vide* cooked vegetables over last five years.

Vegetable Sample	Cooking Treatment	Quality Parameters	Conclusion Remarks	Reference
Potato slices added with rosemary essential oil (REO) Six potato cultivars: Arinda, Elodie, Erika, Fontane, Marabel, Ranomi	Dipping pre-treatments: (i) peanut seed oil with 0.5% (v/v) rosemary essential oil (REO) (ii) peanut seed oil—control *Sous-vide* cooking: 105 °C for 30 min	Ascorbic Acid Total phenols Antioxidant activity (DPPH)	-The addition of REO had no influence on the nutritional content of cooked potato slices. -Ascorbic acid, total phenols and antioxidant activity were noticeably reduced during *sous-vide* cooking. -Although total phenols were well retained after cooking, the antioxidant activity indicated reduction of a mean value of 48%.	Amoroso at al. (2019) [24]
-Cauliflower (*Brassica oleracea* var. *botrytis*)—white rose -Romanesco-type cauliflower (green rose) -Brussel sprouts (*Brassica oleracea* var. *gemmifera*) -Broccoli (*Brassica oleracea* var. *botrytis italica*)	-*Sous-vide* cooking: 90 °C for 45 min (cauliflowers and broccoli) 90 °C for 50 min (Brussel sprouts) -Traditional cooking: unsalted water for 10 min (cauliflowers and broccoli) or 15 min (Brussel sprouts) -Steam cooking: 100 °C for 7 min -Storage: at 2 ± 1 °C for 5 days	The content of dry matter Total ash content Mineral compounds contents (K, Na, Ca, Mg, Mn, Fe, Zn, Cu) Organoleptic properties	-Losses of dry matter were minor in *sous-vide* cooked vegetables, whereas the traditional cooking led to a significant decrease in the dry matter content as compared to the raw material for all vegetable samples. -Treatment type demonstrated a distinct effect on the retention of micro and macro elements in all *Brassica* samples. -In comparison to steam cooking, *sous-vide* allowed higher preservation of the minerals contained in vegetable samples. -*Sous-vide* was the only cooking treatment that resulted in positive organoleptic properties. -The only benefit of boiling in water was the improved process yield.	Florkiewicz and Berski (2017) [64]
-Cauliflower (*Brassica oleracea* var. *botrytis*)—white rose -Romanesco-type cauliflower (green rose) -Brussel sprouts (*Brassica oleracea* var. *gemmifera*) -Broccoli (*Brassica oleracea* var. *botrytis italica*)	-*Sous-vide* cooking: 90 °C for 45 min (cauliflowers and broccoli) 90 °C for 50 min (Brussel sprouts) -Traditional cooking: unsalted water for 10 min (cauliflowers and broccoli) or 15 min (Brussel sprouts) -Steam cooking: 100 °C for 7 min	Microbiological analysis Vitamin C (L-ascorbic acid) content HPLC analysis of glucosinolates (GLS): glucoiberin, progoitrin, sinirgin, glucoraphainin, gluconapin, gluconasturtin, glucobrassicin, 4-metoxyglukobrassicin, neoglucobrassicin	-The use of a lower temperature during *sous-vide* cooking did not affect the quality and microbiological safety of the vegetables. -In comparison to raw vegetable samples, higher concentrations of GLS were determined in steamed vegetables. -Six glucosinolates from 9 identified (glucoraphanin, glucoiberin, progoitrin, gluconapin, glucobrassicin, 4-metoxyglucobrassicin) were found in higher amounts in broccoli prepared by the *sous-vide* method, compared to the samples traditionally cooked. - *Sous-vide* cooking of Brussel sprouts and Romanesco-type cauliflower resulted in greater losses of GLS, in comparison with the traditional cooking. -*Sous-vide* cooking can be an advanced processing method of broccoli intended for direct consumption.	Florkiewicz et al. (2017) [65]
-Cauliflower (*Brassica oleracea* var. *botrytis*)—white rose -Romanesco-type cauliflower (green rose) -Brussel sprouts (*Brassica oleracea* var. *gemmifera*) -Broccoli (*Brassica oleracea* var. *botrytis italica*)	-*Sous-vide* cooking: 90 °C for 45 min (cauliflowers and broccoli) 90 °C for 50 min (Brussel sprouts) -Traditional cooking: unsalted water for 10 min (cauliflowers and broccoli) or 15 min (Brussel sprouts) -Steam cooking: 100 °C for 7 min -Storage: at 2 ± 1 °C for 0, 48 and 120 h	Total phenolic content HPLC analysis of phenolic compounds: sinapic acid, caffeic acid, *p*-coumaric acid, gallic acid, protocatechuic acid Antioxidant activity (ABTS)	-*Sous-vide* appeared to be the most advantageous with regard to caffeic, *p*-coumaric and gallic acids’ stability. -A significant positive correlation was found between antioxidant activity and the total phenolic compounds in raw and thermally treated vegetables. -*Sous-vide* method is considered as the optimal thermal technique for *Brassica* vegetables’ processing with regard to phenolic compounds’ preservation.	Florkiewicz et al. (2018) [66]
Pumpkin (*Cucurbita moschata* cv. Leite)	Cooking with the addition of 0.2% of salt (sodium chloride): Boiling—in water, 8 min Steaming—95 °C, 12 min Microwaving—2450 MHz, 10 min *Sous-vide*—90 °C, 30 min	Ascorbic acid content Total phenols Total flavonoids Total anthocyanins Total carotenoids Color analysis Sensory evaluation	-All cooking methods revealed losses of about 50% for ascorbic acid when compared to raw samples. -*Sous-vide* method affected the reduction of total flavonoids the most (30.27%). -Microwaved samples exhibited the highest level of anthocyanins and carotenoids, whereas the *sous-vide* samples had the most reduced values for both types of pigments with losses of 54.37% and 50.0%, respectively. -For all cooking methods, the total polyphenols content was significantly reduced from 49.68% to 64.94%. -The microwaved pumpkin showed the highest sensory applicability, followed by boiling, steaming and *sous-vide* cooking.	Da Silva et al. (2019) [67]
Asparagus spears (*Asparagus officinalis* L., cv Grande)	Boiling (B): 99.0 ± 1.0 °C for 5 min Steaming (S): 99.0 ± 1.0 °C for 6 min Conventional microwaving (MW): 900 W, 2450 MHz, 1.5 min *Sous-vide* boiling (SV-B): 99.0 ± 1.0 °C for 5 min *Sous-vide* (SV): 80 ℃, 15 min *Sous-vide* microwaving (SV-MW): 900 W, 2450 MHz, 1.5 min	Color parameters Inorganic ion content Soluble sugars Ascorbic acid content Carotenoid content Chlorophyll content Rutin content Sensory evaluation	-MW resulted with the highest weight change, dry weight increase and the greatest total color difference as compared to raw samples. -Although all cooking methods were rated as sensory acceptable, SV-MW showed the best preferences. -SV-MW better preserved nutritive quality and color characteristics in comparison to other cooking methods. -In comparison to raw samples, SV-MW samples displayed increased violaxanthin content by 42%. - Rutin level was not statistically influenced by the cooking methods. -SV-MW was found to be the most suitable method for preservation of asparagus spears.	Gonnella et al. (2018) [23]
22 vegetables: sweet potato, broccoli, beetroot, white onion, red onion, garlic, kale, cauliflower, kohlrabi, red cabbage, carrot, red bell pepper, green bell pepper, yellow bell pepper, parsley root, tomato, leek, celeriac, celery, shallot—onion of Ascalan, spinach, potato	Conventional cooking: 100 ℃, 2–20 min (in dependence of vegetable type) *Sous-vide* cooking: 84 ℃, 30 or 60 min (in dependence of vegetable type)	Antioxidative activity (DPPH, FRAP)	-With no effect of the type of processing (conventional vs. *sous-vide* cooking) and determination method (FRAP vs. DPPH), the antioxidative potential of two vegetables (kohlrabi and red pepper) increased when compared to raw vegetable samples. -*Sous-vide* method resulted in higher antioxidative potential after processing for the case of kale, beetroot, red bell pepper, sweet potato, carrot, cauliflower and kohlrabi as compared to their raw samples. -In comparison to the conventional cooking method, improved antioxidative potential after cooking using the *sous-vide* method was detected for red onion, shallot, broccoli, tomato, parsley root and cauliflower. -When comparing the two types of cooking, the obtained results suggest that an increase in the antioxidant potential was higher for the *sous-vide* technique.	Kosewski et al. (2018) [22]
Tomato powder	*Sous-vide* cooking: 60 °C, 4 h	Amadori compounds (LC-MS/MS) L-ascorbic content Total phenolic content Lycopene content Antioxidant activities (DPPH, ORAC, FRAP, ABTS)	-After the *sous-vide* treatment of tomato powder, losses for the content of L-ascorbic acid (20.35%), total phenolic content (15.98%) and lycopene (10.93%) were determined. -The contents of Amadori compounds in the tomato powder subsequently after *sous-vide* treatment was 2.2 times of that before treatment. -*Sous-vide*-treated tomato powder indicated higher antioxidant activity than that from untreated samples measured by all four assays.	Yang et al. (2020) [44]

* DPPH—2,2,1-diphenyl-1-picrylhydrazyl radical scavenging; FRAP—The ferric reducing/antioxidant power assay; ABTS—2,2’-azino-bis-3-ethylbenzthiazoline-6-sulphonic acid; ORAC—Oxygen radical absorbance capacity.

**Table 3:** Hold time for sufficient thermal 6-log reduction of Listeria monocytogenes for meat, fish or poultry in water baths from 60 to 66 °C [6,104].

Thickness (mm)	Temperature						
	60 °C	61 °C	62 °C	63 °C	64 °C	65 °C	66 °C
5	0:51	0:40	0:31	0:25	0:20	0:17	0:14
15	1:13	1:02	0:53	0:47	0:42	0:38	0:35
25	1:41	1:30	1:21	1:16	1:08	1:03	0:59
35	2:09	1:56	1:46	1:38	1:31	1:26	1:21
45	2:42	2:29	2:17	2:08	2:00	1:53	1:48
55	3:26	3:11	2:58	2:47	2:38	2:30	2:23
65	4:15	3:58	3:43	3:31	3:20	3:11	3:02

**Table 4:** The impact of shelf life on the quality parameters of thermally treated seafood products.

Sample	Heat treatment	Shelf life	Key findings	Reference
Salmon (*Salmo Salard*)	Par-roasting: 300 °C for 3 min *Sous-vide*: 80 °C for 43 min	Anaerobic conditions: 2 °C for 0, 4, 8, 12, 15, 18, 22 and 25 days	-The presence of *Enterobacteriaceae* was only detected on days 18, 22 and 25, but they were always below the minimal detection limit (<10 CFU/g). - *Sous-vide* cooking was found to be efficient in the growth inhibition of *Enterobacteriaceae* in salmon stored at 2 °C for up to 25 days. -The shelf life of the *sous-vide* salmon based on sensory analysis was established at 18 days.	Díaz et al. (2009) [112]
Bonito (*Sarda sarda*, Bloch, 1793	*Sous-vide*: 70 °C for 10 min	4 and 12 °C, 42 days	-The *sous-vide* cooking at 70 °C for 10 min reduced the mesophilic (3.46-log CFU/g) and psychrophilic (2.72-log CFU/g) bacterial counts of the raw material to an undetectable level (<1.00-log CFU/g). -*Sous-vide* bonitos were considered highly acceptable in quality until the 15th day of storage at 12 °C. -The shelf life of cold-stored (4 °C) *sous-vide* bonitos is 28 days.	Mol et al. (2011) [113]
Pirarucu (*Arapaima gigas*)	*Sous-vide*: 60 °C for 9.48 min	2 °C, 49 days	-The dorsal cut of raw pirarucu was the most appropriate cut for developing the *sous-vide* product in comparison to other cuts from raw pirarucu. -On the day 0, the *sous-vide* product reached sensory scores for acceptance ≥ 7 considering the hedonic scale, while on the 49th day, the attributes were scored with 5 on average. -Mesophilic and psychrotrophic anaerobes remained during storage within the acceptable limits.	Pino-Hernández et al. (2020) [114]
Lobster (*Homarus americanus*)	High-Pressure Processing (HPP): 150 MPa or 350 MPa for 10 min at 4 °C *Sous-vide*: 65 °C for 10 min	28 days storage at 2 °C	-Raw lobster pressurized at 350 MPa or *sous-vide* cooked maintained significantly lower microbial counts during storage. -HPP pretreatment did not affect additional shelf life extension for *sous-vide* cooked products. -*Sous-vide* can promote the commercial availability of refrigerated lobster tails in terms of the development of diverse lobster products that are more convenient than live lobsters and have better quality than frozen products.	Humaid et al. (2020) [96]
Atlantic mackerel (*Scomber scombrus*)	*Sous-vide*: 60, 75 and 90 °C for 10, 15 and 20 min	1, 3 and 7 days at 4 ± 1°C	-The strongest effect on the generation of primary and secondary products of lipid oxidation was found to be the duration of chilled storage. -Prolonged chilled storage of *sous-vide* cooked samples had a negative impact on its physicochemical parameters. -*Sous-vide* cooking decreased the firmness of the fish muscle during storage.	Cropotova et al. (2019) [7]

**Table 5:** Literature review on quality indices of *sous-vide* cooked seafood over the last five years.

Seafood Sample	Cooking Treatment	Quality Parameters	Conclusion Remarks	Reference
European sea bass (*Dicentrarchus labrax*)	*Sous-vide* cooking: 90 °C for 10 min The ratio of fish/ingredients was 1:0.002. Addition of: laurel (*Laurus nobilis*) and curcuma (*Curcuma longa*) Storage: 3 ± 1 °C for 60 days	pH Total volatile basic nitrogen (TVB-N) Trimethil amine–nigrogen (TMA-N) Microbial analysis: Total mesophilic aerobic (TMAB) Total psychrophilic aerobic bacteria (TPAB) Members of *Enterobacteriaceae* family Sensory evaluation	-The quality of the *sous-vide* seafood products strongly depends on initial quality parameters (microbiological, chemical and sensory) of the raw material. -All products were microbiologically safe during the storage period (<7.00-log cfu/g). -Aside from *sous-vide* processing, addition of laurel and curcuma could prolong shelf life by approximately 4 and 10 days, respectively. -Higher concentrations of laurel and curcuma could promote extended shelf life, but it might have a negative effect on the sensory perception.	Bolat et al. (2019) [107]
Largemouth bass (*Micropterus salmoides*)	Boiling (BT): 85 °C, 4 min Steaming: 100 °C, 4 min Vacuum boiling (VB): 85 °C, 5 min Vacuum steaming (VS): 100 °C, 5 min *Sous-vide* cooking (SV): 85 °C, 5 min	Color measurements Texture analysis The thiobarbituric acid (TBA) Water migration and distribution Microstructural changes	-VS and SV samples reached desirable quality, displaying more stable protein secondary structure and lower lipid oxidation in comparison to other cooking methods. -Protein structure was less damaged in VB, VS and SV samples compared with other cooked samples. -The VS and SV treatments both showed more immobilized water in comparison to other cooked samples.	Wan et al. (2019) [76]
Atlantic mackerel (*Scomber scombrus*)	*Sous-vide cooking*: 70 and 80 °C for 10 and 20 min with and without use of commercial antioxidants (TR25—rosemary extract and mix of tocopherols and RPT40—rosemary extract, α-tocopherol and ascorbyl palmitate). Storage: 4 ℃, 9 days	Primary and secondary products of lipid oxidation Color parameters	-*Sous-vide* cooking and chilled storage negatively influence oxidative lipid stability in mackerel fillets with respect to primary and secondary lipid oxidation products. -Natural antioxidants positively affect the slower rate of lipid oxidation in cooked samples during chilled storage. -The b* value (yellowness) of the fish flesh significantly correlated with conjugated trienes generated from thermal polymerization of lipids during chilled storage of *sous-vide* cooked fish. -Irrespective of antioxidants used, higher temperature and prolonged cooking times enhanced lipid oxidation in mackerel samples.	Cropotova et al. (2019) [115]
Atlantic mackerel (*Scomber scombrus*)	*Sous-vide* cooking: 60, 75 and 90 °C for 10, 15 and 20 min Storage: 1, 3 and 7 days at 4 ± 1 °C	pH Water content and cook loss Water- and salt-soluble proteins Texture analysis Color parameters Lipid oxidation products	-*Sous-vide* cooking time and temperature showed the minimal influence on the formation of primary and secondary products of lipid oxidation and increase in b* value (yellowness) of the fish samples. -Length of chilled storage led to a significant intensification in oxidation and b* value (yellowness). -Length of chilled storage also had an impact on the structural and textural properties of the fish muscle, leading to a decreased cook loss.	Cropotova et al. (2019) [7]
Atlantic salmon (*Salmo salar* Linnaeus, 1758)	*Sous-vide* cooking: 55, 57.5, 60 and 62.5 °C for 0.08 to 250 min Addition of antioxidants: -non-treated control (C) -0.5% (w/w) citric acid (S) -1% (v/w) oregano essential oil (O) -0.5% (w/w) citric acid + 1% (v/w) oregano essential oil added (OS)	*Listeria monocytogenes* ATCC 7644 inoculation pH value	- The inactivation times of *L. monocytogenes* in control group (C) were Table 4 -The inactivation times of *L. monocytogenes* in control samples (C) were significantly higher than all other treated samples (S, O, OS). -Addition of oregano oil (O), citric acid (S) and their combination (OS) significantly reduced the time required to inactivate *L. monocytogenes*. -Combined treatment (OS) was proven to improve the microbial inactivation at 57.5 and 60 °C better than each of the treatments alone did.	Dogruyol et al. (2020) [108]
Tilapia fillets (*Oreochromis niloticus*)	*Sous-vide* cooking: 60.5 °C for 41 min Addition of antioxidants: T1: Control—no herbs added, T2: added extract of oregano. T3: added extract of rosemary and T4: added extract of basil	Centesimal composition Microbiological analysis Lipid oxidation through Thiobarbituric acid reactive substances (TBARS)	-All samples with added extracts showed significantly higher moisture content, while in control samples, higher protein content was observed. -All samples were in accordance to microbiological standards recommended by legislation. -Control samples exhibited high values from Malondialdehyde (MDA)/kg, demonstrating oxidative rancidity characteristics. -Addition of plant extracts as natural antioxidants prolonged shelf life of *sous-vide* treated tilapia fillets.	Alves et al. (2020) [110]
Cephalic part of tuna (*Thunnus maccoyii*)	*Sous-vide* cooking: (1) 59.5 °C for 13 min (2) 59 °C for 39 min (3) 50 °C for 31 min (4) 50 °C for 62 min	Cooking loss, moisture and crude fat content Thermal protein denaturation (TPD) Color analysis Texture analysis Analysis of ATP-related compounds	-The analysis of TPD showed two peaks at approximately 71 and 48 °C (for actin and myosin, respectively). -Based on obtained results from kinetics analysis, the estimation of TPD under different processing conditions for each protein can be evaluated. -Texture changes were more induced by actin denaturation than myosin denaturation, while myosin denaturation was mostly responsible for changes in color and appearance.	Llave et al. (2018) [116]
